# Factors Contributing to Improved Bone Mineral Density in Patients With Transfusion‐Dependent Thalassemia

**DOI:** 10.1002/kjm2.70279

**Published:** 2026-09-16

**Authors:** Cheng‐Ying Hsu, Hui‐Pin Hsiao, Shyh‐Shin Chiou, Pei‐Chin Lin, Yu‐Mei Liao, Wan‐Yi Hsu, Teng‐Hao Wang, Shuo‐Yu Wang

**Affiliations:** ^1^ Division of Pediatric Genetics, Endocrinology and Metabolism, Department of Pediatrics Kaohsiung Medical University Hospital Kaohsiung Taiwan; ^2^ School of Medicine College of Medicine, Kaohsiung Medical University Kaohsiung Taiwan; ^3^ Graduate Institute of Medicine College of Medicine, Kaohsiung Medical University Kaohsiung Taiwan; ^4^ Division of Pediatric Hematology and Oncology, Department of Pediatrics Kaohsiung Medical University Hospital Kaohsiung Taiwan; ^5^ Center of Applied Genomics Kaohsiung Medical University Kaohsiung Taiwan

**Keywords:** blood transfusion, bone mineral density, diabetes mellitus, osteoporosis, thalassemia

## Abstract

Advances in therapy have extended the life expectancy of patients with thalassemia to near that of the general population; complications such as endocrine disorders and osteoporosis remain prevalent. This study aimed to identify endocrine complications, factors associated with endocrine complications, and changes in bone mineral density (BMD) in patients with transfusion‐dependent thalassemia (TDT). This retrospective study reviewed medical records spanning over 10 years from patients aged 20–50 years with TDT, which included longitudinal follow‐up data on thalassemia and endocrine function. Fisher's exact test was used for contingency tables, and univariate and multivariate binary logistic regression analyses were performed to identify contributing factors. A total of 44 patients were included, 81.8% of whom had at least one endocrine complication; osteoporosis was the most common (56.8%). All patients with a shorter blood transfusion interval (≤ 28 days) had thalassemia major. Shorter transfusion interval was significantly associated with a higher incidence of diabetes mellitus (DM) and female hypogonadism and a lower proportion of patients free of endocrine complications. No factor was statistically associated with osteoporosis in our study. 54.5% patients with osteoporosis had spontaneous improved BMD without pharmacological treatment. Vitamin D and calcium supplementation for > 1 year and absence of DM were linked to improved BMD in univariate analysis, but only absence of DM remained significantly associated in multivariate analysis. In conclusion, this study is based on real‐world clinical data and has several limitations. The incidence of endocrine complications was high among patients with TDT. Osteoporosis was the most common endocrine complication. Absence of DM was associated with greater improvement in BMD of TDT patients with osteoporosis.

## 1 Introduction

Thalassemia is a life‐long genetic disorder characterized by abnormal hemoglobin production, which begins during early childhood. Thalassemia treatment depends on the type and severity of the disease. A common therapeutic approach for thalassemia is blood transfusion [1, 2], with some patients requiring regular and frequent transfusions from childhood to ensure survival and improved quality of life. However, other patients may not require such frequent transfusions [3, 4]. Advancements in modern therapeutic and monitoring tools have substantially reduced the early mortality associated with thalassemia. The average life expectancy for individuals with β‐thalassemia has notably improved, increasing from 17 years in 1970 [5], to a point that by 2011, 89% of patients have survived beyond age 40 [6]. This represents a substantial increase in life expectancy.

As the life expectancy of the patients continues to increase, becoming similar to that of the general population [7], these patients increasingly experience complications as they age [8, 9, 10]. A major, life‐threatening complication associated with thalassemia is iron overload, which occurs due to frequent blood transfusions, increased intestinal iron absorption, and the body's limited ability to excrete excess iron [11]. Excess iron can accumulate in organs such as the liver, the heart, and the endocrine glands, leading to substantial organ damage [12]. Patients with transfusion‐dependent thalassemia (TDT) and those with non‐transfusion‐dependent thalassemia (NTDT) can experience iron overload [13, 14]. Iron overload‐related complications tend to manifest earlier in patients with TDT than in those with NTDT [15]. Consequently, endocrinopathies are prevalent in patients with thalassemia [16, 17]. Endocrinopathies typically become clinically apparent during puberty; however, as patients age, the prevalence of most of these disorders tends to increase [9]. Diabetes mellitus (DM) and low bone mineral density (BMD) are the most common endocrinopathies in patients with hemoglobinopathies [18]. The pathogenesis of diabetes in thalassemia patients is not entirely understood. Some studies suggest that diabetes may develop from iron deposition within pancreatic acinar cells, leading to oxidative stress in β‐cells. Additional risk factors include increased iron accumulation in the liver, hepatitis C infection, and iron overload in the muscles [19, 20]. However, osteoporosis in thalassemia is primarily caused by anemia and iron overload [21], and secondary contributing factors include the chronic use of certain medications (e.g., prolonged corticosteroid therapy), hypogonadism, hyperparathyroidism, chronic liver disease, inflammatory conditions, vitamin D deficiency, renal disease, cardiovascular disease, DM, and dementia [22]. Moreover, patients with thalassemia frequently exhibit reduced height [23].

This study aimed to investigate the association between the frequency of blood transfusions and endocrine function in patients with TDT. We reviewed endocrine complications and osteoporosis and identified their risk factors. During this study, we observed that the BMD of certain patients with osteoporosis improved spontaneously despite the absence of pharmacological treatment specifically targeting osteoporosis. Therefore, we conducted an analysis to investigate the potential factors associated with improved BMD in our TDT population.

## 2 Methods

### 2.1 Study Design and Participants

This retrospective study reviewed medical records spanning over 10 years of patients with TDT at the Division of Pediatric Hematology and Oncology and Division of Pediatric Endocrinology, Department of Pediatrics at our institution. The study was reviewed and approved by our institutional review committee. Eligible participants were men and women aged 20–50 years (as of December 2023) who had received transfusions for over 10 years and had at least 10 years of comprehensive clinical documentation, with records current as of December 2023. Patients were excluded if results from endocrine tests and dual X‐ray absorptiometry (DXA) scans were unavailable.

### 2.2 Data Collection

Data extracted from comprehensive medical records included demographic characteristics (i.e., sex, age, and height), longitudinal follow‐up data on thalassemia and endocrine function, and clinical data on their disease and treatment histories. Furthermore, disease‐related variables included the type of thalassemia, blood transfusion intervals, and history of medication use. Laboratory data included serum ferritin levels, markers of endocrine function, and DXA scans for assessing BMD and identifying osteoporosis.

### 2.3 Endocrine Complication Definition and Group Categorization

Patients requiring an average of ≥ 2 units of red blood cell transfusions per 28‐day period over the 10‐year follow‐up were assigned to the ≤ 28‐day transfusion interval group. Serum ferritin levels were calculated as the average level for each individual over the preceding 10 years. Patients were stratified into two groups based on their average serum ferritin levels over the past 10 years: < 2247.2 pmol/L and ≥ 2247.2 pmol/L. The cutoff value of 2247.2 pmol/L corresponds to 1000 ng/mL, which was selected based on previous clinical guidelines for iron overload management in patients with TDT [24]. Height was defined as the height measured at our outpatient clinic when patients were at least 18 years of age. Standard deviation scores (SDS) for height were calculated using a single reference standard derived from the 50th percentile height of 18‐year‐old men and women in the general Taiwanese population [25].

Patients were categorized as having DM or hypothyroidism if they were receiving or had previously received medications for these conditions. In female patients, hypogonadism was defined as an average estradiol level over 10 years below 73.4 pmol/L, accompanied by amenorrhea or irregular menstrual cycles, before starting hormone replacement therapy. In male patients, hypogonadism was defined as a testosterone level below 5.2 nmol/L before starting hormone replacement therapy.

Osteoporosis was defined as a Z‐score of ≤ −2.0 on at least one DXA scan at any of the following sites: the spine, total hip, or femoral head. For longitudinal BMD comparisons, the same anatomical site was used consistently within each patient. Patients with osteoporosis were further categorized into two groups based on follow‐up DXA assessments. Those whose increased by 0.5 or more from the lowest value [26] or Z‐scores increased to above −2.0 were categorized into the improvement group. The non‐improvement group consisted of patients whose Z‐scores either worsened or increased by less than 0.5. Patients with a history of pharmacological treatment for osteoporosis were excluded from the impr_ove_ment and non‐improvement groups. Normal parathyroid hormone (PTH) levels were defined as 1.2–6.9 pmol/L. Patients who did not meet the criteria for any of the aforementioned endocrine complications were categorized as free of these complications.

The “vitamin D and calcium supplementation > 1 year” group included patients taking vitamin D (daily dose > 800 IU) and calcium supplements for > 1 year after an osteoporosis diagnosis and before any improvement was observed. The “sex hormone supplementation > 1 year” group included female patients with hypogonadism treated with estradiol and medroxyprogesterone for > 1 year and male patients with hypogonadism receiving testosterone supplementation for > 1 year.

### 2.4 Statistical Analysis

Statistical analysis was conducted using SPSS version 27.0 software. Descriptive statistics (mean, standard deviation, range, median for continuous variables, and percentages for categorical variables) were calculated. Fisher's exact test was used to assess the relationship between transfusion interval, ferritin level, osteoporosis, and other variables. Comparisons of age, age at first osteoporosis diagnosis, change in DXA Z‐scores, and the lowest Z‐scores between the improvement and non‐improvement groups were performed using the Mann–Whitney *U* test. Univariate and multivariate binary logistic regression analyses were performed to identify factors associated with improved BMD in thalassemia patients. Due to the limited sample size, only variables that were significant in univariate analyses were included in the multivariate model to reduce the risk of model overfitting. A *p*‐value of < 0.05 was considered statistically significant.

## 3 Results

### 3.1 Patient Characteristics

This study included 44 patients diagnosed with TDT (Table 1), and 21 patients (47.8%) were women. The distribution of thalassemia types was as follows: 79.5% had thalassemia major, 2.3% thalassemia intermedia, and 18.2% α‐thalassemia (all hemoglobin H [HbH] disease). The average serum ferritin level over the past 10 years was 4188.3 ± 5183.8 pmol/L (range 687.9–25,702.0; median 2191.7). The mean blood transfusion interval was 1.16 ± 1.09 months (range 0.5–6; median 0.75), with 79.5% of patients receiving transfusions at intervals less than 28 days. Men had significantly shorter transfusion intervals than women (*p* = 0.029). The mean height SD score (SDS) was −0.93 ± 1.17 (range −3.10 to 1.13; median −1.14). No statistically significant differences were observed between men and women regarding age, type of thalassemia, serum ferritin levels, or height SDS.

**TABLE 1:** Clinical and endocrine characteristics of patients with Thalassemia (*N* = 44).

	Total	Male	Female
	*n* = 44	*n* = 23	*n* = 21
Age, years			
Mean ± SD (Range, median)	35.6 ± 5.9 (22–46, 35)	35.2 ± 6.1 (22–46, 35)	36.1 ± 5.7 (26–46, 37)
Thalassemia type			
α‐thalassemia, HbH disease	8 (18.2%)	2 (8.7%)	6 (28.6%)
β‐thalassemia			
Thalassemia major	35 (79.5%)	21 (91.3%)	14 (66.7%)
Thalassemia intermedia	1 (2.3%)	0 (0%)	1 (4.8%)
Transfusion intervals (months)			
Mean ± SD (Range, median)	1.16 ± 1.09 (0.5–6, 0.75)	0.78 ± 0.51 (0.5–3, 0.75)	1.57 ± 1.39 (0.5–6, 1)
Transfusion intervals of ≤ 28 days	35 (79.5%)	21 (91.3%)	14 (66.7%)
Serum ferritin (pmol/L)			
Mean ± SD	4188.3 ± 5183.8	3665.8 ± 3616.6	4760.2 ± 6535.5
Range	687.9–25,702.0	755.3–14,549.4	687.9–25,702.0
Median	2191.7	2271.5	2022.5
Ferritin of ≥ 2247.2 pmol/L	22 (50.0%)	12 (52.2%)	10 (47.6%)
Height (cm)			
Mean ± SD (Range, median)		167.4 ± 7.0 (156–178, 165)	154.4 ± 5.2 (146–165, 154)
Height SDS			
Mean ± SD (Range, median)	−0.93 ± 1.17 (−3.10–1.13.–1.14)	−0.86 ± 1.31 (−3.10–1.13, −1.32)	−1.00 ± 1.02 (−2.67–1.09, −1.09)
Endocrine complications			
Diabetes mellitus	18 (40.9%)	11 (47.8%)	7 (33.3%)
Hypothyroidism	9 (20.5%)	6 (26.1%)	3 (14.3%)
Hypogonadism	17 (38.6%)	10 (43.5%)	7 (33.3%)
Osteoporosis	25 (56.8%)	14 (60.9%)	11 (52.4%)
None of the aforementioned complications	8 (18.2%)	2 (8.7%)	6 (28.6%)

*Note:* Values are presented as mean ± SD, median (range, median), or number (percentage of group total). No statistically significant sex‐related differences were observed in these characteristics.

Abbreviations: HbH = hemoglobin H disease; SD = standard deviation; SDS = standard deviation score.

### 3.2 Endocrine Complications

Of the 44 patients with thalassemia, 18 (40.9%) were diagnosed with DM. Hypothyroidism was observed in nine patients (20.5%). One woman was diagnosed with hyperthyroidism secondary to Graves' disease, which was considered unrelated to thalassemia. Hypogonadism was observed in 10 men (43.5% of men) and 7 women (33.3% of women); 25 patients had a history of osteoporosis (56.8%). Only eight patients (18.2%) had none of these endocrine complications (Table 1). Univariate analyses identified α‐thalassemia (*p* = 0.002) as a variable associated with the absence of these endocrine complications.

### 3.3 Relationship Between Blood Transfusion Intervals, Ferritin Levels and Endocrine Complications

Patients were categorized into two groups based on their blood transfusion intervals (Table 2): those receiving transfusions at intervals of ≤ 28 days and those with intervals > 28 days. All 35 patients with transfusion intervals of ≤ 28 days had the diagnosis of thalassemia major. A statistically significant association was observed between the transfusion interval and thalassemia type (*p* < 0.001). Compared with patients with transfusion intervals > 28 days, those receiving transfusions at intervals of ≤ 28 days had a significantly higher proportion of patients with DM (*p* = 0.006) and significantly fewer in patients who were free of endocrine complications (*p* = 0.005). Among female patients, a significantly higher proportion of those with transfusion intervals of ≤ 28 days had hypogonadism than those with intervals > 28 days (*p* = 0.047).

**TABLE 2:** Association of blood transfusion intervals, ferritin levels, and osteoporosis with endocrine complications in patients with Thalassemia.

Endocrine complications	Transfusion intervals of ≤ 28 days *n* = 35	*p*‐value	Ferritin of ≥ 2247.2 pmol/L *n* = 22	*p*‐value	Osteoporosis *n* = 25	*p*‐value
Thalassemia type						
α	0	< 0.001	4	1	2	0.060
β	35		18		23	
DM	18	0.006	10	0.760	10	1
Hypothyroidism	9	0.167	4	1	6	0.710
Hypogonadism (total)	16	0.121	10	0.536	11	0.535
Hypogonadism (male)	9	1	6	0.680	5	0.417
Hypogonadism (female)	7	0.047	4	0.659	6	0.063
Osteoporosis	22	0.144	11	0.543	—	—
None of the aforementioned endocrine complications	3	0.005	4	1	9	0.760

*Note:* Values are presented as the number of patients. *p* values were calculated using Fisher's exact test.

No statistically significant association was observed between higher serum ferritin levels (≥ 2247.2 pmol/L) and the presence of endocrine complications (Table 2).

### 3.4 Interrelationship Between Osteoporosis and Other Endocrine Complications

Thalassemia patients with a history of DXA Z‐scores lower than −2.0 (14 men, 11 women) were included in the osteoporosis group. Five men and four women had a history of osteoporosis as their only endocrine complication. Among the osteoporosis group, there were one man and one woman with α‐thalassemia, and 13 men and 10 women with β‐thalassemia (Table 2). The mean blood transfusion intervals of the osteoporosis and non‐osteoporosis groups were 1.01 ± 0.71 months (range 0.5–3, median 0.75) and 1.36 ± 1.45 months (range 0.5–6, median 0.75), respectively. No statistically significant interrelationship between osteoporosis and DM, hypothyroidism, or hypogonadism was detected (Table 2).

### 3.5 Factors Contributing to Improved BMD

Based on serial follow‐up, 14 patients (8 men, 6 women) were categorized into the improvement group (as defined in Methods), whereas 11 patients (6 men, 5 women) were categorized into the non‐improvement group. One man and one woman in the improvement group and 1 man in the non‐improvement group who were receiving denosumab therapy (covered by health insurance) were excluded from this analysis. Others declined referral for osteoporosis therapy or refused such therapy for personal reasons. Therefore, 54.5% (12/22) patients with osteoporosis had spontaneous improvement in BMD without pharmacological treatment. The mean age at first diagnosis of osteoporosis was significantly lower in the improvement group (mean 23.9 ± 4.6 years; range 17–31; median 24) than in the non‐improvement group (mean 28.4 ± 4.8 years; range 21–38; median 27) (*p* = 0.043 by Mann–Whitney *U* test). There was no significant difference in the lowest DXA Z‐scores between the groups (*p* = 0.923). The changes in Z‐scores from the nadir to the most recent measurement were 1.08 ± 0.77 (range 0.4–3.3; median 0.95) in the improvement group and −0.53 ± 0.56 (range −1.6–0.2; median −0.55) in the non‐improvement group. No patients in the improvement group experienced a subsequent BMD decline sufficient to warrant re‐diagnosis with osteoporosis after their initial improvement. Three patients in the improvement group still had osteoporosis, although their DXA Z‐scores had increased by more than 1.0 during follow‐up. Univariate regression analyses demonstrated that the absence of DM and vitamin D and calcium supplementation for > 1 year were significantly associated with improved BMD (*p* = 0.018 and *p* = 0.036, respectively). However, multivariate logistic regression analyses revealed that only the absence of DM was independently associated with improved BMD (*p* = 0.040) (Table 3).

**TABLE 3:** Characteristics of patients with osteoporosis in the improvement and non‐improvement groups, and factors correlated with improved bone mineral density.

	Improvement group *n* = 12	Non‐improvement group *n* = 10	Univariable OR (95% CI)	Univariate *p*‐value	Multivariable OR (95% CI)	Multivariate *p*‐value
Sex						
Male	7 (58.3%)	5 (50%)	1.4	0.696		
Female	5 (41.7%)	5 (50%)	(0.26–7.58)			
Age at first osteoporosis diagnosis of ≤ 30 year	11 (91.7%)	7 (70%)	4.714 (0.41–54.83)	0.215		
Thalassemia type						
α‐thalassemia	2 (16.7%)	0 (0%)	Not estimable	0.999		
β‐thalassemia	10 (83.3%)	10 (100%)				
Transfusion intervals of > 28 days	3 (25%)	0 (0%)	Not estimable	0.999		
Without diabetes mellitus	10 (83.3%)	3 (30%)	11.667 (1.53–89.12)	0.018	13.105 (1.12–153.01)	0.040
Without hypothyroidism	11 (91.7%)	6 (60%)	7.333 (0.66–81.37)	0.105		
Normal PTH level	11 (91.7%)	8 (80%)	2.750 (0.21–35.84)	0.440		
Without hypogonadism	8 (66.7%)	3 (30%)	4.667 (0.77–28.47)	0.095		
None of the aforementioned endocrine complications	6 (50%)	2 (20%)	4.000 (0.59–27.25)	0.157		
Vit D and Ca supplement > 1 year	7 (58.3%)	2 (20%)	12.600 (1.19–133.89)	0.036	14.314 (0.87–235.93)	0.063
Sex hormone supplement > 1 year	3 (25%)	6 (60%)	0.222 (0.04–1.37)	0.105		

*Note:* Values are presented as number of patients (percentage of group total).

Abbreviations: 95% CI = 95% confidence interval; OR = odds ratio.

## 4 Discussion

Our study demonstrated that TDT patients had an extremely high incidence of endocrine complications (> 80%). Patients with transfusion‐dependent (TD) HbH disease experienced a lower incidence of endocrine complications than patients with TD β‐thalassemia major, consistent with previous studies [24, 27]. We also demonstrated that transfusion intervals, rather than serum ferritin levels, were associated with endocrine complications; intervals of ≤ 28 days were associated with a significantly higher incidence of DM and female hypogonadism. Serum ferritin does not reliably reflect the severity of iron overload because it can be influenced by factors such as inflammation, infection, and liver disease [28]. Therefore, additional markers, including serum transferrin saturation, may help improve the assessment of iron overload [28]. Our findings are consistent with previous research suggesting that patients with β‐thalassemia major and prolonged transfusion treatment have a high risk of developing diabetes [29]. A previous study has shown that primary hypothyroidism and hypoparathyroidism typically emerge during the second decade of life, are associated with iron overload, and may be reversible with timely and intensive chelation therapy [30]. Consequently, the early detection and prevention of endocrine dysfunction, specifically through consistent and adequate chelation therapy, are essential for improving the quality of life for these patients.

Among the endocrine disorders observed, osteoporosis was the most prevalent, affecting more than half of our participants, and the youngest patient was diagnosed at age 17. This finding concurs with other studies reporting a high prevalence of osteoporosis in individuals with thalassemia [17]. Although endocrine disorders themselves can contribute to osteoporosis as secondary causes [31], no single endocrine complication was significantly associated with osteoporosis in our patients, and nine patients had osteoporosis as their sole endocrine disorder. Osteopenia and osteoporosis have a complex pathophysiology and are leading causes of morbidity in both male and female young adults with thalassemia [30]. In addition to TDT patients, bone biopsies from NTDT patients also revealed osteoporosis characterized by increased bone resorption, reduced mineralization, and fewer active bone‐forming sites [32]. These findings highlight that early‐onset osteoporosis in thalassemia patients is multifactorial, involving bone marrow expansion, iron overload, and endocrine dysfunction [21, 22, 30].

We found that 54.5% (12 of 22) of patients with osteoporosis achieved BMD improvement through nutritional interventions alone, without pharmacologic therapy for osteoporosis. Our findings provide valuable insights into factors associated with improved BMD. The absence of DM was independently associated with improved BMD in our study, although DM was not the factor associated with osteoporosis. Accumulating evidence has elucidated the deleterious effects of diabetes mellitus on bone health, underscoring a complex interplay of pathogenic mechanisms. Insulin and insulin‐like growth factor 1 (IGF‐1) are pivotal regulators of bone homeostasis, mediating anabolic processes essential for the maintenance of skeletal integrity [33]. Chronic hyperglycemia perturbs bone metabolism by impairing extracellular matrix synthesis and inhibiting mineralization [34]. Prolonged exposure to elevated glucose levels also promotes the accumulation of advanced glycation end products (AGEs) within bone tissue [35]. These AGEs contribute to collagen fiber stiffening and degradation, hinder osteoblast differentiation, and suppress alkaline phosphatase activity [35, 36]. Moreover, chronic inflammation and oxidative stress constitute key drivers of diabetes‐associated bone loss [33]. Elevated levels of pro‐inflammatory cytokines, including tumor necrosis factor‐alpha and interleukin‐6, are consistently observed in diabetic populations and promote osteoclast‐mediated bone resorption, thereby exacerbating skeletal fragility and increasing fracture susceptibility [33, 37]. Therefore, diabetes prevention in patients with TDT has substantial benefits in terms of improving glycemic levels and BMD. Furthermore, the absence of diabetes would serve as an indicator of better self‐healthcare management. This hypothesis is supported by numerous studies showing that adherence to treatment protocols and effective self‐management are strongly associated with improved clinical outcomes in patients with thalassemia [38].

Although not statistically significant in multivariate analysis, we demonstrated that vitamin D and calcium supplementation had probable beneficial effects on improving BMD, which is a cost‐effective therapy, making it particularly relevant in resource‐limited settings. Low serum levels of 25‐hydroxyvitamin D are often observed in patients with thalassemia [21, 39], and combined supplementation with vitamin D and calcium is more effective than either nutrient alone in managing osteoporosis and reducing fracture risk [21, 40]. Moreover, our data suggest that patients diagnosed with osteoporosis at a younger age had higher rates of BMD improvement (61% before 30 years of age vs. 25% after 30 years of age), emphasizing the potential importance of timely diagnosis and early intervention in preventing long‐term skeletal deterioration.

This study is based on real‐world clinical data and has several limitations. These include its retrospective and single‐center design, a relatively small sample size that may have affected the statistical robustness of the analyses and influenced some non‐significant findings, exclusion of individuals without endocrine laboratory data and those under 20 or over 50 years of age, irregular follow‐up with DXA assessments, lack of longitudinal data extending beyond 14 years, and lack of a prospective case–control design. In addition, important potential confounders, including age, sex, chelation therapy, physical activity, and nutritional status, were not fully adjusted for because of the retrospective design and limited sample size. The small sample size also resulted in wide confidence intervals for some odds ratio estimates, indicating limited precision and stability of these estimates. These limitations should be considered when interpreting our findings and highlight the need for larger, multicenter prospective studies to validate our results.

In conclusion, our study found that transfusion intervals, rather than ferritin levels, are associated with DM and female hypogonadism. The incidence of endocrine complications in our TDT patients was high, and osteoporosis was the most prominent endocrine complication and had an early onset. Early BMD assessment, along with nutritional intervention, is recommended for patients with thalassemia. Supplementation with vitamin D and calcium would exert beneficial effects on the improvement of BMD. Furthermore, the absence of DM was associated with improved BMD in our study, although further prospective studies are needed to clarify this relationship.

## Conflicts of Interest

The authors declare no conflicts of interest.

## Data Availability

Data sharing not applicable to this article as no datasets were generated or analysed during the current study.
